# Type 3 Diabetes and Its Role Implications in Alzheimer’s Disease

**DOI:** 10.3390/ijms21093165

**Published:** 2020-04-30

**Authors:** Thuy Trang Nguyen, Qui Thanh Hoai Ta, Thi Kim Oanh Nguyen, Thi Thuy Dung Nguyen, Vo Van Giau

**Affiliations:** 1 Faculty of Pharmacy, Ho Chi Minh City University of Technology (HUTECH), Ho Chi Minh City 700000, Vietnam; nt.trang85@hutech.edu.vn; 2 Institute of Research and Development, Duy Tan University, Danang 550000, Vietnam; tathoaiqui@duytan.edu.vn; 3 Faculty of Food Science and Technology, Ho Chi Minh City University of Food Industry, Ho Chi Minh City 700000, Vietnam; oanhntk@hufi.edu.vn; 4 Faculty of Environmental and Food Engineering, Nguyen Tat Thanh University, Ho Chi Minh City 700000, Vietnam; 5 Graduate School of Environment Department of Industrial and Environmental Engineering, Gachon University, 1342 Sungnam-daero, Sujung-gu, Seongnam-si, Gyeonggi-do 461-701, Korea; 6 Department of Bionano Technology, Gachon Medical Research Institute, Gachon University, 1342 Sungnam-daero, Sujung-gu, Seongnam-si, Gyeonggi-do 461-701, Korea

**Keywords:** Alzheimer’s disease, hypometabolism, type 2 diabetes, type 3 diabetes, insulin resistance

## Abstract

The exact connection between Alzheimer’s disease (AD) and type 2 diabetes is still in debate. However, poorly controlled blood sugar may increase the risk of developing Alzheimer’s. This relationship is so strong that some have called Alzheimer’s “diabetes of the brain” or “type 3 diabetes (T3D)”. Given more recent studies continue to indicate evidence linking T3D with AD, this review aims to demonstrate the relationship between T3D and AD based on the fact that both the processing of amyloid-β (Aβ) precursor protein toxicity and the clearance of Aβ are attributed to impaired insulin signaling, and that insulin resistance mediates the dysregulation of bioenergetics and progress to AD. Furthermore, insulin-related therapeutic strategies are suggested to succeed in the development of therapies for AD by slowing down their progressive nature or even halting their future complications.

## 1. Introduction

Diabetes is a serious, long-term condition with a major impact on the lives and well-being of individuals, families, and societies worldwide. The global diabetes prevalence in 2019 is estimated to be 9.3% (463 million people), rising to 10.2% (578 million) by 2030 and 10.9% (700 million) by 2045 [1]. Population aging is also increasing dramatically throughout the world, especially in developing countries, creating pressures on the health system as well as social security services and policies. In Vietnam, diabetes is projected to be one of the top seven diseases leading to death and disability by 2030 [2,3]. With the increasing prevalence of diabetes, there are approximately 5.76 million people with diabetes currently living in Vietnam. The age-adjusted comparative prevalence of diabetes in the population of Vietnam was approximately 6% in 2017 [2]. Nowadays, many people are familiar with type 1 or type 2 diabetes mellitus, however, there is another form of diabetes that has just recently been identified, known as type 3 diabetes (T3DM). This lesser-known type manifests as insulin resistance within the brain and has major potential to impact neurocognition and contributes to the etiology of Alzheimer’s disease [AD]. AD has already been identified as the sixth leading cause of death in the United States, and the fifth leading cause of mortality in people 65 and older [4]. It has no current cure, but treatments for symptoms are available and research continues. Neurotransmitter deficits, degenerated neurons, synaptic dysfunction, extracellular buildup of β-amyloid (Aβ) and intracellular neurofibrillary tangles (NFT) are the major crude disfigurements present in AD [5]. To produce Aβ peptides of different lengths such as Aβ38, Aβ40, and Aβ42 due to the active enzymatic component of the γ-secretase complex, presenilin 1 (PSEN1), and PSEN2, the amyloid precursor protein (APP) cleaves at several sites within the membrane. Unfortunately, diabetes is following right behind AD as the seventh leading cause of mortality and is projected to affect almost half a billion people by the year 2045 [1]. Both diseases have been recognized to have multifactorial interactions involving both the environment and to a lesser degree, genetics. Yet, insulin insensitivity has been linked to memory deficits, cognitive decline, and many of the characteristic symptoms that have been displayed in AD. At the same time, type 2 diabetes has remained one of the most adjustable risk factors for the development of AD. DM may be classified into four clinical categories: type 1, type 2, type 3, and type 4. Type 1 diabetes (T1D) is mainly due to β-cell destruction, mostly leading to absolute insulin deficiency. The type 2 diabetes (T2D) is due to a progression of insulin secretary defect concomitantly with insulin resistance. Insulin resistance is a common phenomenon, closely associated with obesity, and defined as the inability of target tissues to respond normally to insulin. Insulin resistance typically precedes the onset of type 2 diabetes by several years. T2D is a risk factor for dementia and for AD, the most common type of dementia. T1D is mainly observed in children and young adults, while T2DM is more common among adults and is responsible for 90% of the incidences globally [6]. Some epidemiological studies suggest that insulin resistance increases the risk for dementia and AD, even in nondiabetic populations. 

**Figure 1:**
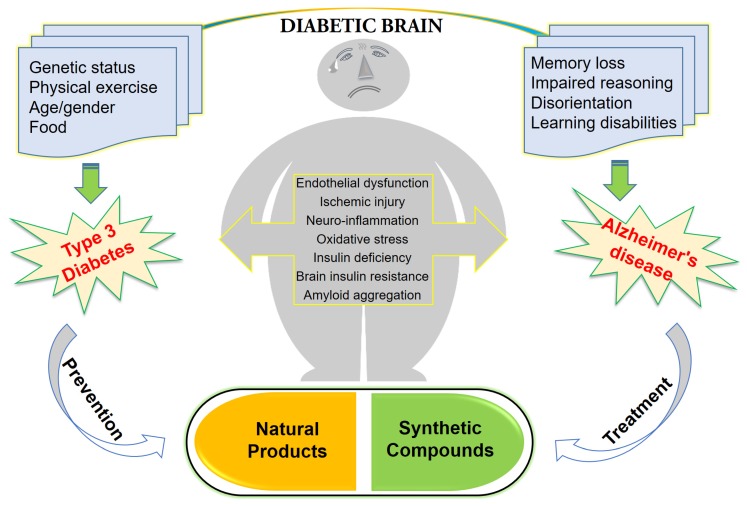
Type 3 diabetes regarding Alzheimer’s disease and its approaches for treatment and prevention.

A recently discovered form has been suggested to be termed type 3 diabetes mellitus (T3DM) by scientists. These scientists have tried to define it as a metabolic syndrome that may lead to abnormalities linked to progressive brain insulin resistance with consequent impairment of central insulin signaling processes, accumulation of neurotoxins, neuronal stress, and resulting in a course of neurodegeneration [7,8]. In vitro and animal studies indicated that insulin resistance can contribute to the pathogenesis of AD through multiple different pathways [7]. Endocrine abnormalities—especially diabetes—are common in AD, which is also regarded as a type of diabetes. Diabetes having an influence on memory processing (recognition and retrieval), morphology of brain (brain atrophy) and synaptic communication is a well demonstrated hazardous aspect that influences the pathology of AD [9]. In addition, the hyperinsulinemia impairment of insulin signaling and insulin resistance are the vital factors that make the sense of keeping insulin at the center stage of both pathologies irrespective of genotype [10]. Many recent studies have indicated that impaired hippocampus insulin signaling impairs the memory and other executive functions, attributing to the decline of insulin signaling and concurrent development of insulin resistance [11,12,13]. This deliberation advocates a strong link between hyperinsulinemia and insulin resistance and the resultant pathologies like T3D and AD [14]. Peripheral insulin resistance leads to decrease insulin signaling in CNS, followed by alteration in brain metabolism. Increased Aβ toxicity, Tau hyperphosphorylation, oxidative stress and neuroinflammation are attributed to central insulin resistance, which leads to neurodegeneration. The work provides the relationship between T3DM and AD based on the fact that both the processing of amyloid-β (Aβ) precursor protein toxicity and the clearance of Aβ are attributed to impaired insulin signaling in the brain. Furthermore, insulin-related therapeutic strategies are suggested to succeed in the development of therapies in AD by slowing down their progressive nature or even halting their future complications. Figure 1 reveals the concept of T3D regarding AD and its approaches for treatment and prevention.

## 2. Insulin and Glucagon Signaling in the Central Nervous System (CNS)

Insulin is a hormone that regulates glucose levels in the blood, is produced by the beta cells of the islets of Langerhans in the pancreas, and consists of two polypeptide chains connected by disulfide linkages. Insulin initiates its action by binding to transmembrane glycoprotein receptors formed by two α and two β-subunits [14]. Insulin binding to α-subunits of the receptors fabricate confirmative alterations that lead to its activation and autophosphorylation of several Tyr residues at β-subunit cytosolic region [15,16]. Autophosphorylated remnants are then acknowledged by the insulin receptor substrates (IRS), out of which IRS-1 and IRS-2 are the two major players and the common intermediaries in insulin signal propagation. IRS is ideal and suitable for the configuration of molecular complexes which mediates intracellular signaling pathways. Insulin and insulin-like growth factors (IGF-1) connect to tyrosine kinase receptors, the insulin receptor (IR) and IGF-1. Insulin binding is highest in the olfactory bulb, cerebral cortex and hippocampus. Furthermore, insulin receptors are also expressive on endothelial cells of the blood–brain barrier and are responsible for transport of insulin and IGF-1 through the blood–brain barrier (BBB) into CNS [17]. While the exact mechanism of how insulin gets into the brain still remains controversial, insulin circulating in the blood can cross the BBB through a receptor-mediated active transport system [17]. This pathway is consistent with studies showing that insulin levels in the cerebrospinal fluid (CSF) increase proportionally with blood insulin after peripheral insulin infusion [15,16,17]. However, the amount of insulin produced in the brain and whether this pool of insulin is physiologically relevant still remains elusive. It is possible that both the centrally and peripherally derived pools of insulin are important for signaling in the brain. 

Insulin and IGF-1 are conferred with functions which are important for neuronal survival and the maintenance of CNS integrity. Insulin receptors and insulin signaling affect glucose homeostasis, neuronal integrity and cognition through influencing several receptor-mediated mechanisms including calcium influx, neurotransmitter build-up and synaptic connections, apoptosis, and neurogenesis [17]. Insulin also regulates expression and levels of GABA, NMDA and AMPA-mediated mechanisms which have a strong influence over long-term potentiation (LTP) and long-term depression (LTD). Furthermore, insulin is crucially involved in expansion and preservation of excitatory synapses [18] and dendritic spine formation through the activation of AKT–mTOR and Ras-related pathways [19,20] which are integral to insulin signaling [21]. Insulin also influences cell survival by modulating apoptotic pathways and the intermediates involved in the apoptotic cascade [22,23]. 

The presence of insulin in the brain was first reported by Havrankova et al. [24] who used radioimmunoassay to determine high levels of insulin in brain extracts. Also, high insulin concentrations had then been reported not only in the human brain but also in several experimental animals [25]. Recently, the production of insulin in the CNS has also been widely studied, and suggestions of possible insulin biosynthesis in the brain have been based on different experimental evidence. Evidence of the presence of insulin mRNA was found in the periventricular nucleus of the rat hypothalamus by in situ hybridization [26]. The molecular mechanisms involved in the production and secretion of insulin in the CNS reveal similarities between beta cells and neurons, particularly in relation to ATP-sensitive K+ channel depolarization that have been demonstrated [27]. This depolarization-induced release of insulin was able to be inhibited by cycloheximide, and was specific for neurons, but not for astrocytes [28]. Interestingly, dysfunctions in the insulin receptors (IRs)-mediated processes might be due to abnormalities in IR activation, lowered insulin availability, and compromised IR-triggered downstream mechanisms resulting in a broad range of brain disorders [29]. Also, IR associated with RNA polymerase II in the nucleus, with striking enrichment at promoters genome-wide have recently been demonstrated [30]. These results reveled that IR interacts with transcriptional machinery at promoters, and identify a pathway regulating genes linked to insulin’s effects in physiology and related diseases [30]. Thus, through influencing any of these pathways, insulin alters the neuronal performance and integrity which may result in defects in learning, memory and other features of AD. Previous studies indicated that brain insulin was equally reduced in AD patients and age-matched controls, indicating that reductions in brain insulin are likely a result of age, not AD [31]. Ultimately, a greater understanding of insulin in the brain relative to the severity of AD and age-matched controls needs to be obtained in order to fully comprehend insulin’s function in healthy and diseased brains. Thus, reduced insulin levels in the CNS can lead to reduced levels of antiamylogenic proteins, and both the overproduction and an impaired clearance of Aβ.

## 3. The Role of Type 3 Diabetes in Glucose Homeostasis

The key to understanding the relationship between diabetes and these other areas begins with the role of energy homeostasis in diabetes. Energy homeostasis is a well-regulated process that depends on the coordination between feeding behavior and energy expenditure. The control of energy homeostasis in humans has received much attention in recent years due to alterations caused by onset of conditions such as obesity and diabetes. There are two distinct features of adult neurons that make them vulnerable to either neuronal cell death or a diseased state such as neurodegeneration or neuronal loss. The first feature is that fully differentiated (adult) neurons are permanently postmitotic cells, which lack regenerative ability [32]. Therefore, when adult neurons are exposed to any cellular stresses such as lack of adenosine triphosphate (ATP) moieties or energy crisis or oxidative stress, they either die or experience apoptosis, or degenerate or cause neuronal degeneration and loss, and thus predispose neurodegenerative diseases [32]. The second important feature is that brain neurons or tissues are highly demanding excitable cells, in which more than 40% of the present ATP is used to keep neurons viable or alive [33]. There are two sources of brain glucose that involve cortical glucose metabolism stimulation through basal insulin levels [34] and astrocytic glycogen conversion to glucose that is stimulated by the activation of glial β-adrenoceptors. The increase in glucose uptake is transported by insulin-sensitive glial glucose transporter type 1 (GLUT1) to the plasma membrane for neuronal use. Therefore, the balanced cellular glucose transportation depends on astrocytes and glucose transporters that are expressed in the brain [35]. 

Moreover, a glucose homeostasis defect might be important in the pathogenesis of T3DM due to impaired glucose uptake as a result of impaired glucose metabolism in the brain. The mechanisms that are involved in glucose transportation abnormalities include brain insulin resistance and intracellular glucose metabolic disturbance. These two abnormalities may contribute to cerebral glucose hypometabolism in T3DM or the brain insulin resistance disease state. A decreased glucose transporters correlated to abnormal hyperphosphorylation of tau in neurodegenerative diseases was reported [36]. Therefore, impairment of insulin signaling not only affects systemic glucose blood levels but also causes various degenerative processes or neuronal cell death or loss [37]. In addition, insulin resistance in T2DM has been defined as “reduced sensitivity in body tissues to the action of insulin” [38]. Similarly, brain insulin resistance can be defined as the failure of brain cells to respond to insulin and its corresponding IRs [39]. Consequently, this leads to insulin deficiency and impaired glucose transport inside the neurons due decreased number of expressed GLUTs in the cell membrane. Furthermore, insulin resistance in the CNS correlates with insulin resistance in the periphery. Therefore, loss of responsiveness to insulin could make neurons more susceptible to neurotoxic insults due to their being devoid of protective effect of insulin [40]. Furthermore, insulin-resistant patients have many increased pathologic features such as apoptosis, neurodegeneration, and the resultant decline in cognition. 

The desensitization of the neuronal insulin receptor in brain insulin resistance, similar to the process in T2DM, may play a key role in causing T3DM and its future complications [41]. Besides, T2DM is a metabolic syndrome characterized by insulin resistance, which is also a pathological feature of neurodegeneration or neuroendocrine disorder or T3DM [34]. Thus, glucose homeostasis plays a role in T3DM pathogenesis. Brain glucose uptake or metabolism is impaired in T3DM. Therefore, the combination of T2DM and neurodegenerative brain diseases may be considered as this new classification of diabetes, called T3DM or a neuroendocrine disorder.

## 4. Type 3 Diabetes and Aβ Protein Pathology

Amyloidosis is a pathological condition which consists of the accumulation of fibrillary proteins, characterizing by extracellular amyloid deposits with a clinical variability depending on the affected tissue. Recently, there has been newly emerged evidence regarding the relationship between the pathogenesis of AD and insulin resistance. It is important to consider T2DM as a risk factor essential for the formation of deposits of amyloid-β in patients’ brains with dementia. There was a toxic cycle between continuous insulin exposure and Aβ accumulation inside the neurons [42]. According to Farris et al., insulin degrading enzyme (IDE) regulates the levels of insulin, Aβ protein, and amyloid precursor protein (APP) intracellular domain in vivo [42]. This study showed that a rat model of T2DM of mutant IDE was associated with hyperinsulinemia and glucose intolerance, as hallmarks of T2DM and T3DM or brain insulin resistance. This implies that IDE hypofunction may underlie or contribute to some forms of T3DM and T2DM and provide a mechanism for the recently recognized association among hyperinsulinemia, diabetes, and neurodegeneration or neuronal loss [42]. Therefore, in normal subjects, IDE reduces Aβ, regulates insulin and also degrades APP intracellular domain (AICD). Thus, there was a regulatory relationship among insulin, IDE and Aβ. In the case of brain insulin resistance, insulin possibly failed to stimulate the clearance of Aβ, which permits its buildup inside the neurons causing neurodegeneration or neuronal loss, as hallmarks of T3DM or brain insulin resistance [42]. There is a debate about T3DM and brain insulin resistance as to whether it is a consequence or a cause of abnormal Aβ expression and protein processing [43]. In terms of the concept of T3DM being a consequence, Aβ toxicity may cause insulin resistance in the brain. The Aβ disturbs insulin signaling by competing with insulin on its receptors [44], reducing the surface expression of IRs, and reducing the insulin affinity to its relative receptors, and interfering directly with phosphatidylinositol-4, 5- bisphosphate 3-kinase (PI3K)/Akt activation, causing a blockade of its signaling and leading to impaired survival signaling, increased activation of GSK-3β activity, and increased hyperphosphorylation of tau [45]. 

**Figure 2:**
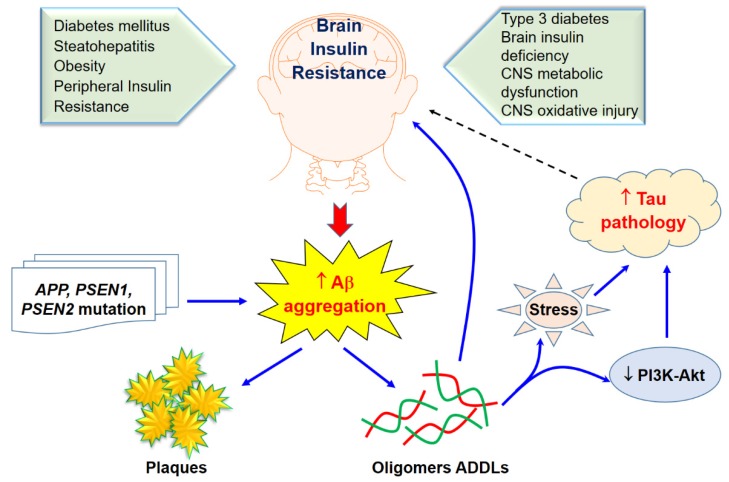
Brain insulin resistance and Aβ aggregation and its toxicity. Solid arrows indicate the interactions of Aβ aggregation on brain insulin resistance through sone potential pathways while tau pathology would likely effect of brain insulin as revealed in a dasher arrow.

On the other hand, in terms of the concept of T3DM being the cause, the brain insulin resistance with oxidative stress and neuroinflammation may cause Aβ accumulation, as shown in Figure 2. The studies that incorporate this concept claim that insulin stimulation may increase or accelerate trafficking of Aβ from the Golgi network to the plasma membrane. Therefore, insulin may activate Aβ extracellular excretion and, at the same time, inhibit its intracellular accumulation by activating its degradation by the insulin-degrading enzyme (IDE) [46]. Thus, impaired insulin signaling can disturb both APP processing and Aβ clearance [47]. This leads to increased neurotoxic effects of Aβ on neurons, resulting in possible neurodegeneration and neuronal cell death. T2DM and AD patients have similar amyloid beta deposits both in pancreas and in the brain. Several researchers have suggested this new pathology should be addressed as T3D [48,49,50,51]. Some of the target receptors of T2DM such as the insulin-like growth factor 1 (IGF-1) and peroxisome proliferator-activated receptor gamma (PPARG) are also involved in the regulation of the expression and phosphorylation of tau protein [51]. 

## 5. Type 3 Diabetes Regarding Alzheimer’s Disease 

Insulin resistance in AD and diabetes can lead to hyperinsulinemia, thereby, saturating insulin-degrading enzymes (IDE) for insulin and Aβ degradation. Recently, many studies indicated that the incidence of AD is higher in T2D patients and obese individuals, implying common mechanisms driving these disorders [10,52,53]. Insulin resistance could be a main feature which is shared among diabetes, obesity, and AD [54]. The neuronal glucose uptake may not depend on insulin totally, thus the concept of insulin resistance in the brain is more related to impaired insulin signaling pathways. The malfunction of insulin signaling pathways and resultant state of hypometabolism observed are considering among factors in altered bioenergetics that connects AD and T2D [55]. The insulin resistant state could lead to compromised neuron functions and cognitive skills, accompanied by an extreme rise in insulin and relatively declined insulin activity in the periphery as important predictors of T2D [56,57]. Consequently, this leads to the development of neuritic plaques, hippocampal atrophy, cognitive performance and lower cerebrocortical glucose metabolism which may closely correlate with memory impairments [50]. A previous study revealed that increased p-Ser312IRS1 manifested in prodromal AD patients that sustained these alterations a decade ago as AD patients [58], suggesting that insulin resistance in AD develops years before clinical manifestations and that neural-derived exosomes carry potential for early AD diagnosis. Due to lack of insulin response, down regulation of insulin receptors, reduced binding of insulin receptors or faulty activation of the insulin signaling cascade cause the defective brain insulin signaling in AD and T2D. The major consequence of this altered cascade is the decreased neuronal glucose uptake that is manifested as impaired neuroplasticity, neurotransmitter deficits, collapse of bioenergetics mechanism and initiation of fateful inflammatory cascade. Overall, the consequences of impaired insulin signaling are attributed to impaired metabolism in the brain that may lead to brain malfunction, providing possible explanations for the connection between diabetes, obesity, and AD [11], as shown in Table 1. 

**Figure 3:**
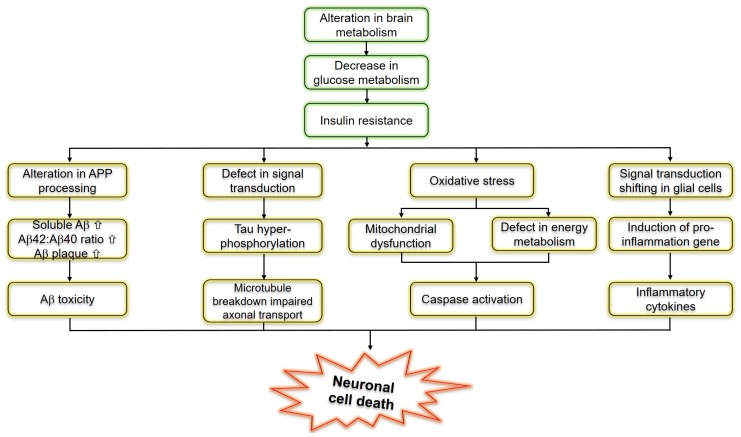
Schematic representation of molecular pathways linking insulin resistance and Alzheimer’s disease.

Insulin resistance or dysfunction of insulin signaling is a universal feature of T2D, due to altered glucose metabolism and its interdependence on cell death pathways form the basis of linking T3D with AD, as shown in Figure 3. T3D occurs when neurons in the brain become unable to respond to insulin, which is essential for basic tasks, including memory and learning. Some researchers believe insulin deficiency is central to the cognitive decline of AD. Dysfunctional insulin pathways and resistance of insulin is a status of receptor dysfunction, altered receptor expression, deviations in receptor binding and malfunctioned events in the phosphorylation chain or the altered activities related to kinases involved in phosphorylation. At the molecular level, a cell senses insulin through insulin receptors, with the signal propagating through a signaling cascade collectively known as the PI3K/Akt/mTOR signaling pathway. Recent studies suggested that the pathway operates as a bistable switch under physiologic conditions for certain types of cells, and insulin response may well be a threshold phenomenon [13,59,60]. The pathway’s sensitivity to insulin may be blunted by many factors such as free fatty acids, causing insulin resistance. It also is based on the finding that insulin resistance may be reversed rapidly by exposing cells to mitochondrial uncouplers, electron transport chain inhibitors, or mitochondrial superoxide dismutase mimetics [61,62].

Interestingly, impaired insulin signaling is present in several transgenic and nontransgenic mouse models of AD. Some previous clinical studies have reported that AD patients could have glucose intolerance, suggesting a bidirectional relationship between the two conditions [63,64]. Reduced levels of IRS-1 associated to the membrane of hippocampal extracts [65] and a decreased activation of IRS-1 and PI3K in the hippocampus and cortex were observed in ten-month-old mice [66]. Markers of insulin resistance were also reported in the hypothalamus of APP/PS1 mice [67] since the IRS-1 phosphorylated in serine 616 in the hippocampus at nine months of age was higher than that of the control group [68], and increased levels of IRS-1 phosphorylated in serine 636 and 312 in the frontal cortex at 13 months [69] were also demonstrated. In combination with peripheral insulin resistance, there was also a report of an increased inhibitory phosphorylation of IRS-1 in serine 612 in the hippocampus of five-month-old tg2576 mice [66]. Remarkably, the central infusion of AβOs lead to peripheral insulin resistance, which was further observed in the APP/PS1 and in the 3xTgAD mouse models of AD [70]. To confirm these concepts, further evidence is still required to investigate the mechanisms whereby AD affects the diabetic phenotype. T3D regarding AD and its approaches for treatment and prevention using naturally synthetic compounds, as shown in Figure 2.

In addition, in the wake of the worldwide increase in T2DM, a major focus of research aims to understand the signaling pathways impacting this disease. Insulin signaling regulates glucose, lipid, and energy homeostasis, predominantly via action on liver, skeletal muscle, and adipose tissue. Cell signaling pathways can be described by a list of biomolecular reactions which occur between the pathway components. T2DM associated with impaired insulin and insulin-like growth factor-1 (IGF1) signaling (IIS) is a risk factor for cognitive impairment and dementia including AD [71]. Importantly, systemic heterozygous inactivation of IGF1R (IGF1R+/−) or neuronal deletion of IGF1R (nIGF1R−/−) could improve the survival in the Tg2576 mouse model of AD while reducing behavioral impairment and Aβ accumulation [72]. Reduced IRS2 signaling throughout the body or in the brain prolongs life span [73] may lead to systemic reduction of IRS2 (IRS2−/−), improves cognitive function, and reduces Aβ deposition and premature mortality in Tg2576 mice with normal blood glucose levels [72,74]. Hence, more recent animal studies have revealed that a reduction in intracellular signaling mediated by IGF1R-IRS2 signaling but not the IR cascade in the CNS exerts neuroprotective effects in AD animal models [71].

## 6. Therapeutic Approaches to Type 3 Diabetes in Alzheimer’s Disease 

Insulin resistance is well known as an essential feature of T3D, therefore treatment strategies for T3D, particularly those aimed at improving insulin sensitivity, may also benefit those patients at risk for AD at the early stages. Due to the overlapping yet distinct pathological features among diabetes, insulin resistance and cognitive decline, multitargeted drug therapies along with lifestyle interventions are also explored [75] from the perspective of research in the pharmaceutical industry, including nutraceuticals, antioxidant activity [76], polyphenols, omega-3 fatty acids as well as the brain–gut connections [77]. 

Among nutraceuticals produce, a brain-permeable compound, curcumin is able to target abnormal protein aggregates [78]. Curcumin may also thwart “proapoptotic signaling pathways in primary hippocampal neuron cultures”. Previous research has also shown the benefit of metformin in mice when coupled with curcumin and piperine supplementation, particularly regarding enhanced insulin sensitivity, signaling, and better systemic glucose tolerance [78], promising natural substances for AD patients. However, the anti-inflammatory benefits of fruits and vegetables have been widely publicized for decades, particularly regarding antioxidant action in reducing inflammatory damage [79]. Rodent research has linked various vegetables and fruits as protective “against cognitive and brain neuropathology from dietary oxidative stress” due to innumerable bioactive constituents such as carotenoids, antioxidant vitamins, polyphenols and flavonoids [80]. A various families of flavonoids have been suggested to be the potential therapeutic implications via in vivo models [81]. This has significant potential to advance our understanding of proactive approaches toward preventing AD and inhibiting progression. The essential role of omega-3 fatty acids in brain development and maintenance has been well recognized, particularly in the past ten years, yet only recently “have their effects on brain aging been explored” [82]. Diets rich in omega-3 fatty acids and naturally low in omega-6fatty acids may hold the key for nutritional therapy for AD patients [83]. The ketogenic diet may even diminish and clear beta amyloid plaques within the brain, while convalescing damaged mitochondria and reducing universal inflammation [84]. New research has shown that glycated APOE4 protein and faulty insulin signaling leads not only to impaired energy transport for brain tissues, but also impaired lipid transportation, mainly cholesterol [84,85]. APOE4 accounted for approximately 20% of the general population and >50% among Alzheimer’s cases, is responsible for interrupting how the brain processes insulin [86]. The gene and the peripheral insulin resistance caused by the high-fat diet together induced insulin resistance in the brain [87]. The APOE4 protein produced by the gene can bind more aggressively to insulin receptors on the surfaces of neurons than its normal counterpart, APOE3. APOE4 goes on to do lasting damage to brain cells. After blocking the receptor, the sticky APOE4 protein begins to clump and becomes toxic [87]. Furthermore, once the protein enters the interior of the neuron, the clumps get trapped within the cell’s machinery, impeding the receptors from returning to the neuron surface to do their work. The insulin signal processing gets increasingly more impaired, starving brain cells. There is no pharmaceutical intervention that has ever existed that has been more potent in improving overall vasculature throughout the body, than exercise [88]. This also has extensive implications for AD patients and type 2 diabetics due to increases in quality of life, neurochemical messaging within the brain, restorative power over insulin resistance, and the ability to clear Aβ plaques in certain individuals [88]. The concept of the gut–brain axis, the bidirectional communication between gut and brain, contributing significantly to the pathogenesis of AD that has been supported by many experimental and clinical studies [77]. Representatives of some compounds and drugs for the treatment or prevention of T3D regarding AD progression are presented in Table 2.

## 7. Conclusions

The relationship between T3DM and AD is based on the fact that both the processing of AβPP and the clearance of Aβ are attributed to impaired insulin signaling in the brain. Additionally, it focuses on the molecular mechanism of brain insulin resistance that may involve either increased serine phosphorylation of IRS-1 protein (i.e., IRS-1 inhibition) and elevated degradation of IRS protein as common pathological mechanisms, including aggregation of toxic Aβ plaques, tau hyperphosphorylation and autophagy. Increasing the knowledge and awareness of the term T3D has the potential to pave the way for disease treatment, prevention and possibly even deliver a cure. Currently, there have been no particular treatments with established efficacy in counteracting cognitive decline or AD, so the implications of identifying AD as a disorder with an etiology rooted in faulty insulin signaling and irregular energy pathways could be critical in disease management. While the specific mechanisms between AD and all forms of diabetes remain convoluted and unclear, which subsequently may have devastating socioeconomic impacts on public health and healthcare systems, T3D has the potential to provide a plethora of proactive and therapeutic strategies to current patients. For now, it seems that the testing of more anti-T3D drugs with beneficial effects against cognitive impairment has a certain promising future.

## Figures and Tables

**Table 1:** Causal model for the potential associated with between T3D and AD.

Upstream Risk Factors	Metabolic Precursors	Pathways		Subclinical Pathology	Disease Outcome
Social factors: stress, low socioeconomic status, certain ethnic and racial groups	Obesity Visceral Adiposity	**Vascular Processes** Blood pressure and hypertension Hyperlipidemia Apolipoprotein E	Cerebral blood flow Atherosclerosis	Amyloid precursor proteins	Alzheimer’s disease
Poor diet: high in calories, fat and sugar, low in fiver		**Inflammatory/Oxidative processes** Inflammation Oxidative stress Endothelial function		Neurofibrillary Tangles Amyloid B deposits	
Physical inactivity Genetics and family history	Hyperglycemia Hyperinsulinemia	**Metabolic processes** Insulin resistance Insulin-degrading enzyme Peroxisome proliferative-activated receptors			
Early childhood exposures in utero and birth weight	Brain and hippocampal atrophy White matter hyperintensities				

**Table 2:** Summary of representative of preclinical and clinical studies on the efficacy of antidiabetic, insulin-sensitizing drugs on multiple aspects of AD pathology.

Compound	Potential Pathway	Study Design	Reference
DA5-CH	Reduces tau phosphorylation and normalizes theta rhythm	Injected intracerebroventricula (ICV), streptozotocin on rat	[89]
DA-JC1	Antagonizing circadian rhythm disorders induced by Aβ_31–35_	ICV, amyloid(31–35) AD model	[90]
DA5-CH	Improved of hippocampal synaptic plasticity and activation of the PI3K/AKT signaling pathway	APP/PS1 mouse model of AD	[91]
DA-CH3	Reduced ER stress and apoptotic signaling, reduced amyloid plaque load in the brain	APP/PS1 mouse model of AD	[92]
Insulin	Prevention of Aβ oligomer induced synapse loss and insulin receptor reduction, amelioration of PKR-mediated ER stress	Rat hippocampal neuronal cultures	[93,94]
Insulin	AD patients that are not ε4 carriers have reduced sensitivity to insulin, effecting cognitive performance	AD patients homozygous or not for the ApoE-ε4 allele and normal subjects intravenously injected	[95]
Insulin	Improved verbal memory in MCI AD ε4-subjects after acute insulin administration, but not in ε4 carriers	AD patients homozygous or not for the ApoE-ε4 allele, MCI patients and most subjects intranasally administrated	[96,97]
Insulin	Chromic intranasal insulin doses enhanced selective attention, retention of new information and functional status of MCI and early AD subjects	AD patients, MCI patients and normal subjects intranasally administrated	[98]
Insulin	Only women presented improved working memory after treatment	Healthy men and woman intranasally administrated	[99]
Liraglutide	Reduction of tau phosphorylation; protection of insulin reception and synapse loss in a c-AMP dependent manner	Cynomolgus monkeys ICV with Aβ oligomer	[100]
Liraglutide	Improvement of memory deficits in novel object recognition test and fear conditioning	Swiss mice injected ICV with Aβ oligomer	[100]
Liraglutide	Restored memory deficits in object recognition test and Morris water maze; enhanced LTP; reduced microglial activation; diminished Aβ plaque load	APP/PSEN1 mice	[101,102]
Exendin-4	Decrease in the inhibitory phosphorylation of Ser312IRS1, Ser66IRS1 of INK, while restoring activating Tyr465 IRS1 phosphorylation	Rat hippocampal neural cultures	[69]
Exendin-4	Improvement of spatial memory in the Morris water maze; reduced amyloid plaque LOAD	APP/PS1 mice	[69]
Exedin4- Liraglutide	eIF2α phosphorylation reduction	Rat hippocampal neural cultures, APP/PS1 mice, cynomolgus monkeys injected ICV with Aβ oligomer	[94]
GLP-1 Exendin-4	Reduction of neural excitotoxicity	Rat hippocampal neural cultures, rats injected on the basal nucleus with ibotenic acid	[103]
Rosiglitazone	Reversal of memory deficits in objects recognition test and the Morris water maze; Aβ levels reduction	AD transgenic mice J20 line	[104]
